# Lipoamide Attenuates Hypertensive Myocardial Hypertrophy Through PI3K/Akt-Mediated Nrf2 Signaling Pathway

**DOI:** 10.1007/s12265-024-10488-9

**Published:** 2024-02-09

**Authors:** Hongjuan Cao, Lina Zhao, Yao Yuan, Chunyan Liao, Weidan Zeng, Aiyue Li, Quanfeng Huang, Yueyao Zhao, Yubing Fan, Liu Jiang, Dandan Song, Sha Li, Bei Zhang

**Affiliations:** 1 Guizhou Medical University, Guiyang, Guizhou Province China; 2 Department of Ultrasound Center, Affiliated Hospital of Guizhou Medical University, Guiyang, Guizhou Province China

**Keywords:** Lipoamide, Cardiac hypertrophy, PI3K/Akt, Oxidative stress

## Abstract

The process of myocardial hypertrophy in hypertension can lead to excessive activation of oxidative stress. Lipoamide (ALM) has significant antioxidant and anti-inflammatory effects. This study aimed to investigate the effects of ALM on hypertension-induced cardiac hypertrophy, as well as explore its underlying mechanisms. We evaluated the effects of ALM on spontaneously hypertensive rats and rat cardiomyocytes treated with Ang II. We found that ALM was not effective in lowering blood pressure in SHR, but it attenuated hypertension-mediated cardiac fibrosis, oxidative stress, inflammation, and hypertrophy in rats. After that, in cultured H9C2 cells stimulated with Ang II, ALM increased the expression of antioxidant proteins that were decreased in the Ang II group. ALM also alleviated cell hypertrophy and the accumulation of ROS, while LY294002 partially abrogated these effects. Collectively, these results demonstrate that ALM could alleviate oxidative stress in cardiac hypertrophy, potentially through the activation of the PI3K/Akt-mediated Nrf2 signaling pathway.

**Graphical Abstract:**

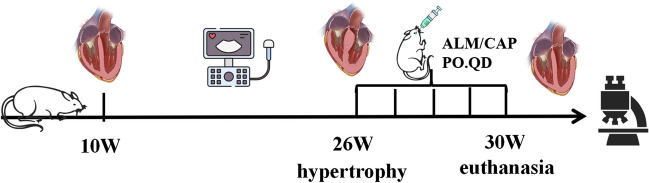

## Introduction

Primary hypertension is a disease characterized by an increase in systemic arterial blood pressure of unclear cause. The long-term increase in arterial blood pressure is followed by various degrees of damage to various organs, of which the heart is the direct organ of blood pressure increase, and left ventricular wall thickening is the earliest change in the heart [1]. In a state of chronic hypertension, the pressure load on the heart increases, and growth factors stimulate cardiomyocyte hypertrophy and myocardial interstitial fibrosis, causing left ventricular hypertrophy and dilation [2]. It has been suggested that left ventricular hypertrophy is considered an independent risk factor for sudden death, ventricular arrhythmias, myocardial ischemia and heart failure [3].

There are studies reporting the numerous mediators that have been found to be involved in the pathogenesis of cardiac hypertrophy affecting gene transcription, calcium handling, protein synthesis, metabolism, autophagy, apoptosis [4, 5], fibrosis [6, 7], oxidative stress, and inflammation [8, 9], among which oxidative stress has been shown to be one of the key factors contributing to hypertension [10–12]. Oxidative stress refers to a state of imbalance between oxidation and reduction processes in the body. When the body is exposed to various harmful stimuli, such as elevated blood glucose and smoking, excessive ROS are produced and insufficient clearance of ROS causes an increase in the accumulation of ROS in the body, which in turn causes oxidative stress to various cellular components. Long-term sustained hypertension can cause excessive production of ROS in myocardial tissues, which exceeds its own clearance capacity and leads to myocardial tissue damage [13]. As one of the pathogenic mechanisms of hypertensive heart disease, reducing the production of ROS and increasing the activity and content of antioxidant substances are important entry points for the treatment of hypertensive heart disease [14, 15]. The phosphatidylinositol-3 kinase (PI3K)/protein kinase B (Akt) signaling pathway plays an important role in the regulation of many physiological processes by activating downstream effector molecules involved in cell cycle transition and cell proliferation [16]. In addition, regulation of the PI3K/Akt pathway which can reduce myocardial hypertrophy has been demonstrated [5, 17]. Nuclear factor erythropoietin-2-related factor 2 (Nrf2) is a major transcription factor that is expressed in most tissues and plays an important role in the amplification of enzyme-related antioxidant pathways in the myocardium. Increased ROS production and PI3K-Akt signaling can activate Nrf2 [18]. Nuclear translocation occurs when Nrf2 is activated to upregulate heme oxygenase 1 (HO-1) expression [19] and initiates the production of antioxidant enzymes such as catalase (CAT), glutathione (GSH), and superoxide dismutase (SOD), and there are numerous studies demonstrating the cardioprotective effects of Nrf2 [20, 21]. Han et al.’s study showed that chlorogenic acid protects MC3T3-E3 cells against oxidative stress through the PI3K/Akt-mediated Nrf2 signaling pathway to induce the expression of HO-1 and antioxidant substances, suggesting that Nrf2 may be a downstream signal target of PI3K/Akt [22].

The current clinical use of angiotensin-converting enzyme inhibitors, angiotensin II receptor blockers, and beta-blockers for hypertensive disease can lower blood pressure and alleviate cardiac dysfunction by reducing myocardial hypertrophy. These drugs inhibit neuroendocrine signals that also act on cardiomyocyte growth, myocardial hypertrophy, and heart failure. However, recent studies have shown that myocardial hypertrophy and the development of congestive heart failure remain inevitable [23, 24]. Therefore, it is particularly important to reduce the occurrence of myocardial hypertrophy in hypertension [25–27]. Lipoic acid is a natural nutrient with a good antioxidant effect and is used in clinical practice for the prevention and treatment of diabetic neuropathy [28] and neurodegenerative diseases [29], In addition, it has been shown that lipoic acid can be used as an antioxidant to treat cell damage caused by oxidative damage to mitochondria [30] as well as heart and kidney damage caused by hypertension [31]. Lipoamide (ALM) is a neutral amide derivative of lipoic acid, which has not been sufficiently studied, but has been shown to resist oxidative stress-mediated neuronal cell damage [32] and prevention of mitochondrial dysfunction [33, 34]. However, the effect of ALM in the treatment of hypertensive cardiac hypertrophy and its mechanism have not been studied. Hence, the aim of this study was to investigate the therapeutic effect of ALM in spontaneously hypertensive rats and to verify whether its therapeutic effect is via the PI3K/Akt pathway.

## Experimental Method

### Experimental Animal Models and Handling

Six 9-week-old male Wistar rats (WT) and eighteen 9-week-old male spontaneously hypertensive rats (SHR), weighing 200–250 g, were purchased from the Experimental Animal Centre of Guizhou Medical University, and all animal experimental protocols were approved by the Animal Ethics Committee of Guizhou Medical University (No. 1900572). All animals were fed water ad libitum and housed at 20–25 °C. At 26 weeks, SHR blood pressure was at a stable high value. At this point we started daily administration of ALM (150 mg/kg/d) [35] and captopril (CAP) (50 mg/kg/d) [36] gavage treatment. Each group consisted of 6 rats. The treatment ended after 4 weeks. Body weight was measured weekly, and rats were executed at the end of treatment after pentobarbital sodium anesthesia [37] and blood sampling through the femoral artery, and cardiac tissue was removed and weighed to calculate the heart weight to body weight ratio.

### Cell Culture

Rat cardiomyocyte (H9C2) cells were purchased from the American Type Culture Collection (ATCC). They were cultured in Gibco high glycemic medium (DMEM) containing 10% fetal bovine serum and placed in an incubator (5% CO2, 37 °C). Before establishing the myocardial hypertrophy model, H9C2 cells were pretreated with PI3K inhibitor (LY294002) at 10 μM for 1 h [38] and co-treated with 1 μM angiotensin II (Ang II) [5] combined with different concentrations of ALM for 48 h. The groups were control group, Ang II group, Ang II + 10 μM ALM group, Ang II + 50 μM ALM group, AngII + 100 μM ALM group, AngII + 150 μM ALM group, AngII + 200 μM ALM group, and AngII + 10 μM LY294002 + 100 μM ALM group.

### Echocardiography

Rats were weighed and anesthetized with 1% pentobarbital sodium. Transthoracic 2-dimensional (2D), M-mode, and Doppler echocardiographic studies were performed with Mylab 50 (Esaote, Italy) using a high-resolution transducer (SL3116) with a frequency of 22 MHz. M-mode images were obtained to measure intraventricular septal thickness during diastole (IVSd) and left ventricular internal dimension (LVID) during diastole (LVIDd) and systole (LVIDs). The following parameters of systolic function were also calculated: left ventricle ejection fraction (LVEF), left ventricle fractional shortening (LVFS), left ventricular end systolic volume (LVESV), and left ventricular end diastolic volume (LVEDV). For each measurement, data from at least three consecutive cardiac cycles were averaged.

### Blood Pressure Measurement

Systolic blood pressure (SBP) and diastolic blood pressure (DBP) were assessed on a regular basis in the tails of conscious rats using a noninvasive computerized tailcuf system (Kent Scientific Corporation, CT, USA). Each rat was placed in a heated tube (38 °C) for 10–15 min, after which its body temperature was monitored to increase. Each rat was measured three times, and each set of rats was measured concurrently.

### Immunoblotting

Protein samples were extracted from rat cardiac tissue, and protein concentrations were determined by BCA method, and the amount of protein spiked in each protein sample was calculated to be consistent. The protein samples were separated by gel electrophoresis according to the conventional method, transferred and closed at room temperature; primary antibodies Bax (Proteintech 1:2000 dilution), Bcl2 (Proteintech 1:1000 dilution), Fibronectin (Proteintech 1:1000 dilution), Col-III (Proteintech 1:1000 dilution), Cleaved-caspase3 (1:1000 dilution), Nrf2 (Proteintech 1:1000 dilution), HO-1 (Proteintech 1:1000 dilution), PI3K (Proteintech 1:5000 dilution), Akt (Cell Signaling Technology 1:1000 dilution), *p*-PI3K (Cell Signaling Technology 1:2000 dilution), and *p*-Akt (S473) (Cell Signaling Technology 1:1000 dilution) were added and incubated at 4 ℃ overnight; secondary antibodies were added and incubated at room temperature for 1 h. The protein bands were analyzed by the ImageJ image processing system with β-actin (Proteintech 1:10,000 dilution) as the internal reference, and semi-quantitative analysis was performed.

### Histological Analysis

After echocardiographic examination, the rats were deeply anesthetized with pentobarbital sodium solution and executed by blood sampling through the femoral artery, and the heart tissues of each group were dissected and collected, fixed and then dehydrated, and embedded and sectioned (5 μm) to produce myocardial histopathological sections for hematoxylin–eosin (H&E) and Masson staining, and the myocardial histopathological changes were observed by light microscopy.

### WGA Staining

The tissue sections underwent dewaxing in xylene and hydrating through a graded series of ethanol to water and were incubated for 1 h with WGA dying in the dark. Phosphate-buffered saline was used to washing the slides 3 times. The cell nucleus was stained with 4′,6-diamidino-2-phenylindole (DAPI) for 10 min. The slides were sealed with antifade mounting medium and then observed using a fluorescence microscope.

### ELISA

Myocardial tissues were collected and stored at − 80℃, 200 mg of myocardial tissues was grinded and prepared as homogenate, centrifuged at 3000 r/min for 15 min, and the supernatant was extracted, and the levels of IL-1β, IL-6, and TNF-α in myocardial tissues were measured by enzyme-linked immunosorbent assay (ELISA) kit (Mlbio, Shanghai China) according to the manufacturer’s instructions. A microplate absorbance reader (Thermo, USA) was employed to measure the absorbance at 450 nm. Standard curves were applied to calculate the analyte concentrations.

### RNA Extraction and Quantitative Real-Time PCR (qPCR) Analysis

qPCR was used to detect the mRNA expression levels of hypertrophic markers ANP and BNP in Rats. Total RNA was obtained from tissue by the Trizol method, and then cDNA was synthesized using a reverse transcription instrument according to the manufacturer’s instructions. Real-time polymerase chain reaction was performed using SuperReal PreMix (Bio-Rad, USA), and qPCR SYBR Green Master Mix (YEASEN, Shanghai China) was performed. The sequences of the primers used were as follows: ANP forward, 5′-GAGAGTGAGCCGAGACAGCAAAC-3′ and ANP reverse, 5′-GAAGAAGCCCTTGGTGATGGAGAAG-3′; BNP forward, 5′-CCAGTCTCCAGAACAATCCACGATG-3′ and BNP reverse, 5′-GCCTTGGTCCTTTGAGAGCTGTC-3′; and β-actin forward, 5′-TGTCACCAACTGGGACGATA-3′ and β-actin reverse, 5′-GGGGTGTTGAAGGTCTCAAA-3′. β-Actin was used as the internal control. Primers were obtained from Sangon Biotech (Shanghai, China).

### Related Index Testing

LDH, SOD, MDA, and CAT detection kits were purchased from Jian Cheng Institute of Biotechnology (Nanjing, China). Weigh 0.02 g of tissue, add saline at the ratio of weight (g): volume (mL) = 1:9, and make 10% homogenate by homogenizer, centrifuge to extract the supernatant. For the detection of MDA, the homogenate was used for the determination; for the detection of SOD, the sample was diluted 50 times with saline on the basis of 10%. The MDA content, SOD activity, LDH activity and CAT activity in rat heart tissues were measured according to the instructions of the LDH, SOD, MDA and CAT detection kits.

### CCK8

Cell viability was analyzed by the Cell Counting Kit-8 (CCK8, Beyotime, China) according to the manufacturer’s protocols. Cells were seeded and cultured at a density of 5 × 10^3^/well in 100 μL of medium in 96-well microplates (NEST, USA). Then, the cells were treated with various concentrations of ALM (10, 50, 100, 200, and 400 μmol/L). After treatment for 48 h, 10 μL of CCK-8 reagent was added to each well and then cultured for 2 h. All experiments were performed in triplicate. The absorbance was analyzed at 450 nm using a microplate reader (Thermo, USA) using wells without cells as blanks. The proliferation of cells was expressed by the absorbance.

### Measurement of Cell Surface Area

Cells in each group were fixed with 4% paraformaldehyde; thereafter, cells were washed with 0.1%Triton X-100 and stained with Actin-Tracker Red-Rhodamine (1:150 dilution, Beyotime, China). A confocal microscope was used to photograph the cells after nuclear blocking with DAPI staining.

### Reactive Oxygen Assay

Cells were inoculated in Confocal dishes (Biosharp, China) and treated with different drugs. 50 μM of reactive oxygen fluorescent dye H2DCFDA (MCE, China) was added to each well, and cells were incubated for 1 h in the incubator and washed with PBS, and the ROS production in each group was observed under a laser confocal microscope after nuclear blocking with DAPI staining.

### Statistical Analysis

All data were expressed as mean ± standard deviation (SD) and were analyzed by SPSS Version 27 software. Differences between groups were determined and analyzed using one-way analysis of variance (ANOVA) followed by the LSD, using GraphPad Prism 8.0 (GraphPad Soft-ware, San Diego, CA, USA) to draw statistical charts. Statistical significance was defined as *P* < 0.05.

## Results

### ALM Attenuates Cardiac Dysfunction in Spontaneously Hypertensive Rats

**Fig. 1:**
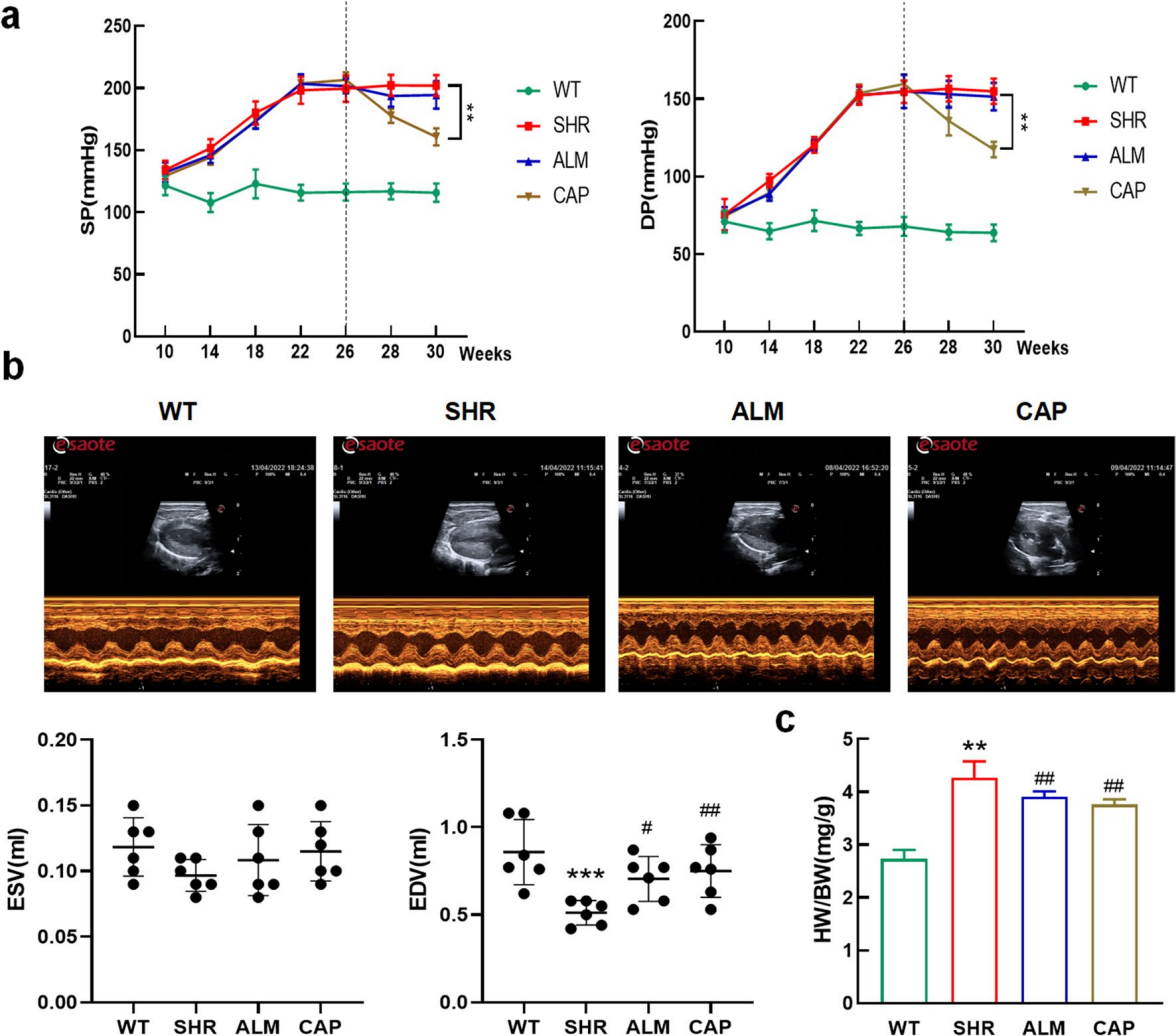
ALM attenuates cardiac dysfunction in spontaneously hypertensive rats. **a** Systolic blood pressure (SBP) and diastolic blood pressure (DBP). **b** Ultrasound detection of LVESV and LVEDV. **c** HW/BW for each group. **P < 0.01 and ***P < 0.001 vs. the WT group. ^#^*P* < 0.05 and ^##^*P* < 0.01 vs. the SHR group

After the blood pressure in spontaneously hypertensive rats had stabilized, left ventricular myocardial hypertrophy was detected by ultrasonography, and treatment with ALM and CAP was started. Blood pressure was checked weekly during treatment. Blood pressure changes in rats after treatment as shown in Fig. 1a. In SHR rats, both systolic and diastolic arterial pressures were higher compared to WT rats, and ALM did not effectively affect blood pressure, while CAP effectively attenuated systolic and diastolic blood pressure. The SHRs revealed ventricular wall hypertrophy and cardiac dysfunction as evidenced by an increased IVSd and decreased EF, FS, and LVIDd. ALM and CAP treatment ameliorated these changes with reduced IVSd and increases in EF, FS, and LVIDd, compared with the SHR group (Table 1). In addition, changes in LVESV and LVEDV were measured, and the results indicated that compared to the WT group, the SHR group showed a significant decrease in LVEDV, suggesting impaired diastolic function in the hearts of hypertensive rats. Compared to the SHR group, treatment with ALM and CAP resulted in an increase in LVEDV, indicating that both drugs can improve cardiac dysfunction (Fig. 1b); however, this effect was not significant in end-systolic volume. Hypertension significantly increased the HW/BW ratio of rats, which ALM attenuated as shown in Fig. 1c. Thus, these results suggested that ALM alleviated hypertension cardiac dysfunction.

**Table 1:** Effect of ALM and CAP on cardiac structure

	WT	SHR	ALM	CAP
IVSd	1.70 ± 0.13^#^	2.20 ± 0.22	1.96 ± 0.24^#^	1.96 ± 0.15^#^
LVIDd	7.23 ± 0.57^#^	6.02 ± 0.31	6.75 ± 0.46^#^	6.89 ± 0.52^#^
LVIDs	3.59 ± 0.26	3.37 ± 0.12	3.48 ± 0.27	3.55 ± 0.23
LVEF	85.95 ± 2.88^#^	80.49 ± 3.72	84.51 ± 2.57^#^	84.32 ± 3.37^#^
LVFS	50.29 ± 3.49^#^	43.92 ± 3.60	48.38 ± 3.02^#^	48.31 ± 4.12^#^

Values are means ± SD

*WT* wistar rats, *SHR* spontaneously hypertensive rats, *ALM* Lipoamide, *CAP* captopril, *IVSd* interventricular septal wall thickness at diastole, *LVIDd* left ventricular internal dimensions at diastole, *LVIDs* left ventricular internal dimensions at systole, *LVEF* left ventricle ejection fraction, *LVFS* left ventricle fractional shortening

^#^*P* < 0.05 vs. the SHR group

### ALM Reduces Hypertension-Mediated Myocardial Hypertrophy

**Fig. 2:**
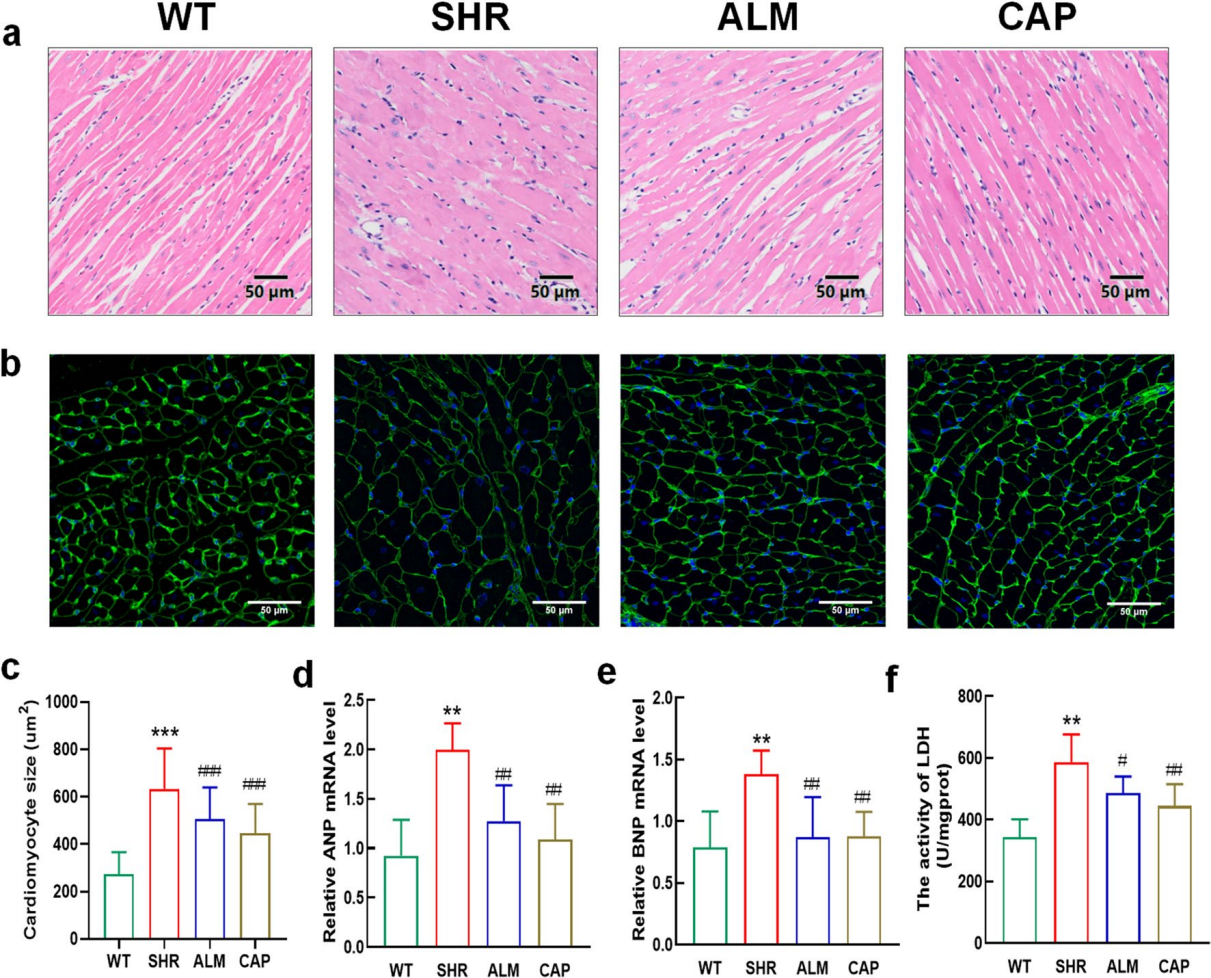
ALM reduces myocardial hypertrophy due to hypertension. **a** Representative image of the left ventricle by H&E staining. **b** WGA staining of ventricular tissue in each group. **c** The cardiomyocyte area of the four groups. **d, e** The mRNA levels of ANP and BNP. **f** LDH activity in cardiac tissue. **P < 0.01 vs. the WT group. ^#^*P* < 0.05 and ^##^*P* < 0.01 vs. the SHR group

Subsequently, H&E staining revealed disordered myocardial cells, increased cross-sectional area, and reduced intercellular space in the cardiac tissues of rats in the SHR group, all of which were alleviated by ALM and CAP treatment (Fig. 2a). The cross-sectional areas of cardiomyocytes were visualized by WGA staining. The results further demonstrated that the cross-sectional areas of cardiomyocytes were increased in SHRs, while ALM and CAP reduced these increases (Fig. 2b, c). Furthermore, qPCR results indicated that ALM and CAP treatment significantly improved the upregulation of ANP and BNP mRNA expression in the ventricular tissue of SHR (Fig. 2d, e). Examination of LDH activity in myocardial tissue showed that both ALM and CAP reduced the increase in LDH activity induced by myocardial injury in SHR rats (Fig. 2f), leading to the conclusion that myocardial injury was reduced after treatment. These results demonstrate that ALM and CAP treatment significantly improved cardiac hypertrophy caused by hypertension.

### ALM Mitigates Hypertension Myocardial Tissue Apoptosis and Fibrosis

**Fig. 3:**
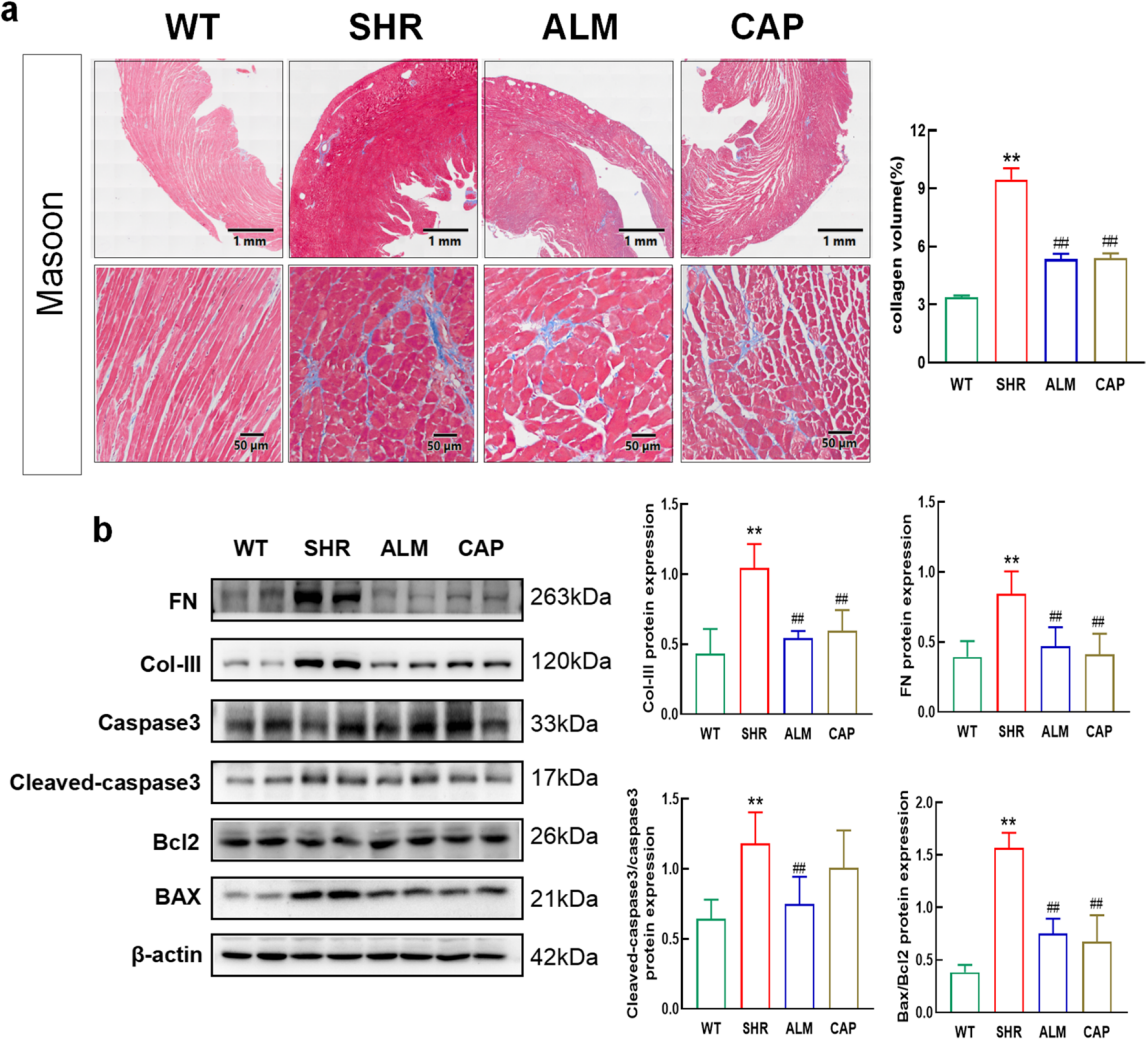
ALM attenuates excessive apoptosis and fibrosis in cardiac tissue. **a** Representative image of the left ventricle by Masson staining. **b** Western blot and quantification of FN, Col-III, Bcl2, Bax, and cleaved-caspase3. **P < 0.01 vs. the WT group. ^##^*P* < 0.01 vs. the SHR group

The fibers in the perivascular and interstitial tissue appeared dark blue in Masson staining. Compared with the WT group, the SHR group showed an increased area of blue staining with darker color, while the ALM and CAP groups exhibited a reduced area of dark blue staining (Fig. 3a). The expression of Bcl-2 decreased in the hearts of hypertensive rats, while the expression of Bax, FN, Col-III, and cleaved-caspase3 increased. ALM treatment mitigated these effects (Fig. 3b). Meanwhile, the attenuation of cleaved-caspase3 protein expression in the CAP group was not significant. The results indicated an increase in fibrosis and apoptosis in the SHR group, which was alleviated after treatment with ALM and CAP.

### ALM Reduces Oxidative Stress and Inflammation in the Heart of SHRs

**Fig. 4:**
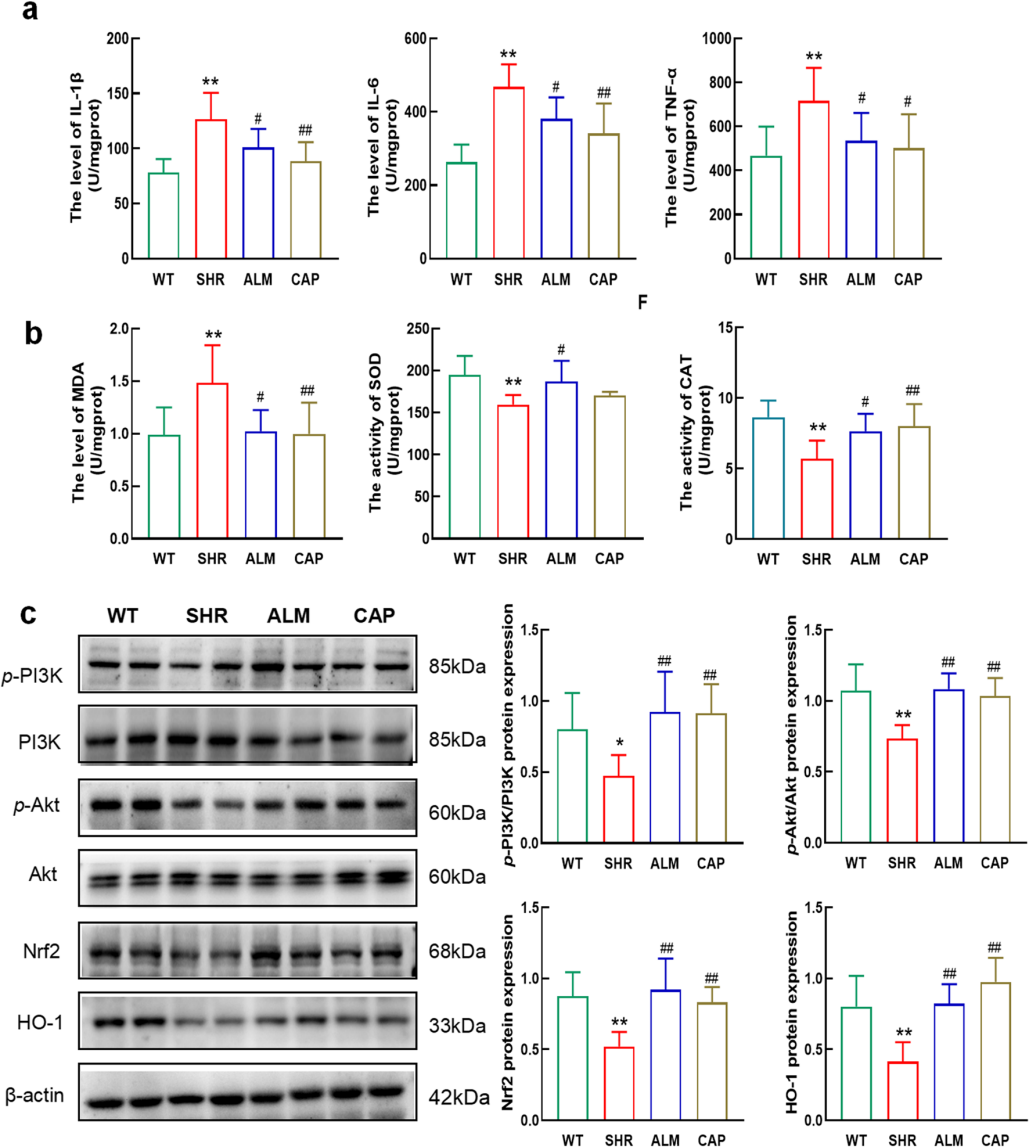
ALM reduces the level of oxidative stress, inflammation in the heart. **a** The levels of IL-1β, IL-6, and TNF-α in vivo. **b** The level of MDA and the activity levels of CAT and SOD. **c** Western blots and quantification of Nrf2, HO-1, *p*-PI3K/PI3K, and *p*-Akt/Akt. *P < 0.05 and **P < 0.01 vs. the WT group. ^#^*P* < 0.05 and ^##^*P* < 0.01 vs. the SHR group

The expression of IL-1β, IL-6, and TNF-α in the hearts of SHR rats was significantly elevated compared to the WT group, indicating an increase in the level of inflammation. After treatment with ALM and CAP, the levels of IL-1β, IL-6, and TNF-α in the hearts of SHR rats decreased (Fig. 4a). In addition, compared to the WT group, the SHR group showed an increase in MDA content and a decrease in SOD and CAT activity, indicating tissue damage from oxidative stress. After treatment with ALM or CAP, the MDA content in the hypertensive rat hearts decreased, and the SOD and CAT activities increased (Fig. 4b), indicating the restoration of cardiac antioxidant capacity. Subsequently, the results of immunoblot analysis showed that compared to the WT group, the expression of Nrf2, HO-1, p-PI3K/PI3K, and p-Akt/Akt was downregulated in the SHR group (Fig. 4c), while after ALM treatment, the levels of Nrf2, HO-1, p-PI3K/PI3K, and p-Akt/Akt increased. These results indicate that the therapeutic effect of ALM on hypertensive cardiac hypertrophy manifests as a reduction in oxidative stress and inflammation levels.

### ALM Improves Oxidation Levels Through the PI3K/Akt Pathway

**Fig. 5:**
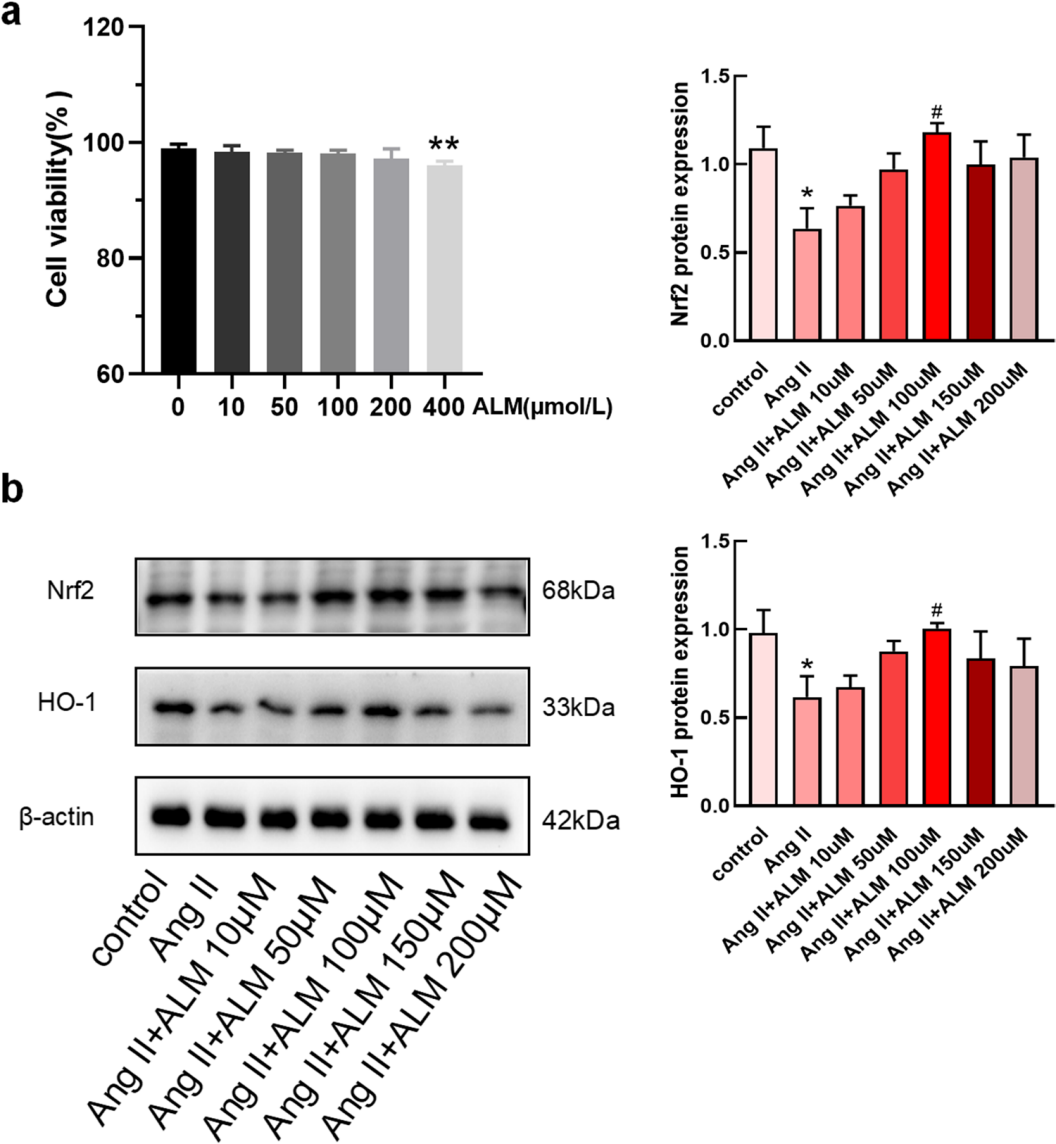
Effect of ALM on the viability of H9C2 cells. **a** CCK8 detects the inhibition of H9C2 proliferation in different concentration treatment groups. **b** Western blot and quantification of Nrf2 and HO-1 after treatment of cells with different concentrations of ALM. *P < 0.05 and **P < 0.01 vs. the control group. ^#^*P* < 0.05 vs. the Ang II group

**Fig. 6:**
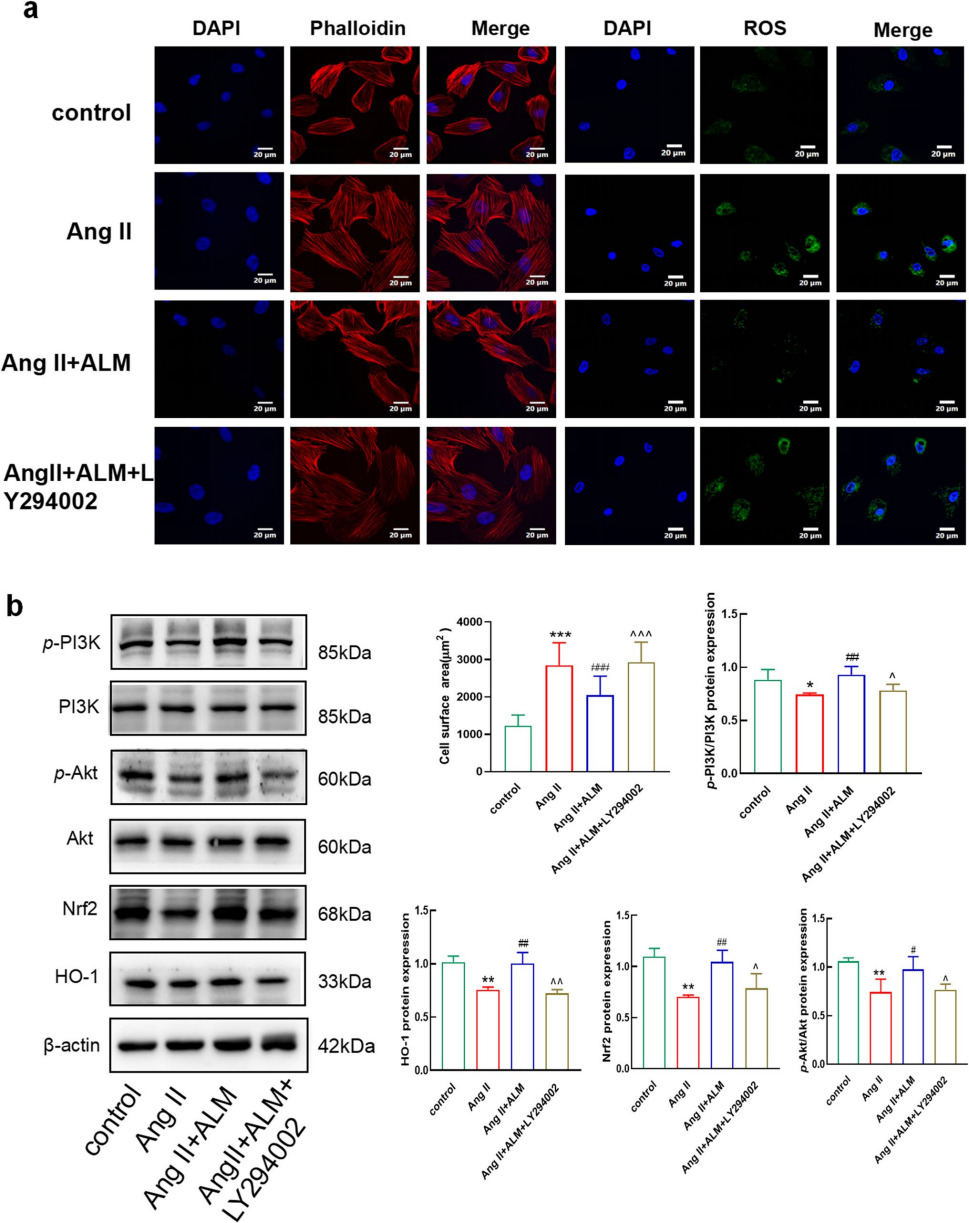
ALM attenuates cardiac oxidation levels through PI3K/Akt. **a** Red-Rhodamine staining was performed to determine cell size and quantitative analysis of intensity of green fluorescence by Reactive oxygen fluorescence staining. **b** Western blot and quantification of p-PI3K/ PI3K, p-Akt/ Akt, Nrf2 and HO-1. *P < 0.05 and **P < 0.01 vs. the control group. ^#^*P* < 0.05 and ^##^*P* < 0.01 vs. the Ang II group. ^^^*P* < 0.05 and ^^^^*P* < 0.01 vs. the Ang II + ALM group

We treated normal myocardial cells with different concentrations of ALM (0, 10, 50, 100, 200, and 400 µM) to observe cell viability and select an appropriate ALM dosage. Cell viability was determined using the CCK8 assay (Fig. 5a). The results showed that cell viability was inhibited at a dosage of 400 µM ALM compared to the control group (0 µM ALM), and there was no significant effect on the cells after 48 h of treatment with other dosages. Therefore, we selected five concentrations of ALM (10 µM, 50 µM, 100 µM, 150 µM, and 200 µM) to treat H9C2 cells exposed to Ang II and found that the expression levels of Nrf2 and HO-1 were most effectively restored at an ALM concentration of 100 µM (Fig. 5b). This concentration was chosen for subsequent experiments. To observe the therapeutic effect of ALM in vitro, we cultured H9C2 cells exposed to Ang II. Compared to the control group, the cell surface area of the Ang II group significantly increased, while the cell surface area decreased after ALM treatment compared to the Ang II group (Fig. 6a). We noticed that the cell surface area of the LY294002 group was greater than that of the ALM group (Fig. 6a). To further investigate the protective effect of ALM on H9C2 cells stimulated by Ang II, we tested whether inhibiting the activity of PI3K would negate the improvement in oxidative stress by ALM. The results of ROS determination showed that ALM reduced ROS levels in Ang II-treated H9C2 cells, but its effect was weakened in the presence of LY294002 (Fig. 6a). This suggests that the inhibition of PI3K activity by LY294002 partially reversed the therapeutic effect of ALM. We performed immunoblot analysis to detect the phosphorylation of PI3K and Akt, as well as the non-phosphorylated proteins. Ang II reduced the phosphorylation of PI3K and Akt, while ALM increased their phosphorylation. Neither Ang II nor ALM treatment affected the total levels of PI3K or Akt (Fig. 6b). Immunoblot analysis of downstream regulatory factors showed that LY294002 treatment reduced the upregulation of Nrf2 and HO-1 induced by ALM (Fig. 6b). These results indicate that ALM protects H9C2 cells from Ang II-induced injury by releasing the inhibition of the PI3K/Akt pathway.

## Discussion

ALM is a neutral amide derivative of the known antioxidant drug lipoic acid, which has been shown in several studies to have anti-inflammatory and antioxidant effects [39, 40]. Lipoic acid regulates metabolism by activating the PI3K/Akt signaling pathway [41]. It has been shown that ALM has better antioxidant properties than lipoic acid [42], ALM can resist H_2_O_2_-induced oxidative damage in PC12 cells, and this protection is exerted by activating Nrf2 and its downstream detoxifying and antioxidant enzymes [32] and the neuroprotective effect of ALM in an animal model of Parkinson’s disease [43]. However, it remains unknown whether ALM can treat myocardial hypertrophy due to hypertension. We examined the protein expression levels of Nrf2 and HO-1 in the heart and found that Nrf2 and HO-1 expression decreased due to myocardial hypertrophy. Correspondingly, the activity of antioxidant enzymes such as SOD and GSH decreased, indicating that the antioxidant capacity of the heart was reduced. The restoration of Nrf2 and HO-1 expression and antioxidant content after ALM treatment indicated that ALM improved the antioxidant capacity of the heart.

In the current study, blood pressure was found to be decreasing continuously in the CAP group by weekly blood pressure testing, while the ALM group did not show significant improvement in systolic or diastolic blood pressure. Analysis of echocardiographic results revealed that the septum was significantly thickened and cardiac function was decreased in the SHR group compared to the WT group, which was improved by ALM and CAP treatment. We found that ALM reduced the levels of IL-1β, IL-6, and TNF-α in the myocardial tissues of hypertensive rats and significantly improved tissue inflammation levels. Several studies have shown that oxidative stress caused by ROS production is closely related to the level of inflammation, and ROS are important signals promoting tissue inflammation [44, 45]. This study found that ALM improved the antioxidant capacity of tissues and reduced the accumulation of ROS in Ang II-treated H9C2 cells; therefore, we suggest that ALM attenuates inflammation probably by reducing ROS production and antagonizing oxidative stress. These observations supported that ALM treatment could confer beneficial effects against hypertensive pathological cardiac remodeling.

The central downstream effector of the PI3K-Akt signaling pathway, which regulates cell survival, remains incompletely defined. Bcl-2 and apoptosis proteins (such as BAX and BAK) are key regulators of apoptosis, and the cell suicide program is essential for the development and tissue homeostasis. Previous reports indicate that cell survival depends largely on the Bcl-2 family of antiapoptotic and apoptosis regulators. There are studies showing that Bcl-2 is involved in cardiac hypertrophy as a key downstream effector of the PI3K-Akt signaling pathway [46]. WB experiments were used to detect apoptosis-related proteins, which revealed that ALM attenuated the elevated levels of apoptosis caused by hypertension, and these results showed that ALM may inhibit apoptosis through the PI3K/Akt pathway.

There are studies suggesting that oxidative stress is implicated in the pathogenesis of cardiac fibrosis both directly and via its involvement in cytokine and growth-factor signaling [47]. In this study, fibrosis-related protein levels were detected by WB, as well as Masson staining. These results indicated that ALM attenuated fibrosis levels in hypertensive myocardial hypertrophy, which may be accomplished by reducing oxidative stress.

Many studies have reported that long-term cardiac hypertrophy increases the likelihood of heart failure, but the treatment of cardiac hypertrophy has not been well defined, because of the obscure molecular mechanism. Multiple myocardial hypertrophy-related signaling pathways have been identified, such as the PI3K-Akt, calcineurin/NFAT, and MAPK pathway [48]. The PI3K-Akt pathway plays a central role in regulating metabolism, glucose uptake, proliferation, and protein synthesis, all of which have the single goal of promoting cell survival. Nrf2, an important transcription factor regulating the antioxidant system, is considered a novel therapeutic target for the treatment of myocardial hypertrophy [49]. There are studies showing that Nrf2 may be a downstream signal target of PI3K/Akt because treatment protects cells against oxidative stress through the PI3K/Akt-mediated Nrf2 signaling pathway to induce the expression of HO-1 and antioxidant substances [22, 50], and several studies have suggested that the PI3K/Akt signaling pathway and its downstream signaling molecule Nrf2 are closely associated with cardiomyocyte hypertrophy [51, 52]. By detecting *p*-PI3K and *p*-Akt protein expression in the myocardial tissue, we found that the PI3K/Akt signaling pathway was inhibited in myocardial hypertrophy, and ALM attenuated this effect. Next, we verified whether ALM could regulate the PI3K/Akt signaling pathway to attenuate oxidative damage in vitro experiment. It is known that pressure overload can activate the renin-angiotensin system (RAS) and induce the release of Ang II. In this study, we assessed the effects of ALM treatment on H9C2 cells following ANG II exposure. We found that ALM treatment restored the increase in cell surface area caused by Ang II stimulation of cardiomyocytes via actin staining. H9C2 cells was pretreated with LY294002 to inhibit the phosphorylation of PI3K, and we observed that cell surface area and accumulation of ROS were increased and the protein expression levels of Nrf2 and HO-1 were decreased when compared to H9C2 cells treated with ALM. Based on these results, we suggest that oxidative stress damage due to abnormalities in the PI3K/Akt pathway is involved in the development of hypertensive myocardial hypertrophy, and ALM attenuates oxidative stress by activating the PI3K/Akt signaling pathway.

During the study, we also found that the positive control group (CAP) showed a decrease in cleaved-caspase3/caspase3 expression relative to the hypertensive group but did not show statistically significant values, a result that we speculate may be due to insufficient duration of CAP treatment or an error in the assay. Similarly, superoxide dismutase activity rebounded in the CAP-treated group relative to the hypertensive group, but the comparison between the two groups was not statistically significant, thus further suggesting that the possibility of error is relatively small and the possibility of insufficient duration or dose of CAP treatment is more likely. The shortcoming of this study is that our treatment time was not long enough and the blood pressure of the experimental animals did not return to the normal range. In the follow-up study, we will further investigate the mechanism of myocardial hypertrophy in hypertension treated with ALM. As the most oxygen consuming organ, the heart is rich in mitochondria, and whether the treatment of cardiac hypertrophy with ALM affects the mitochondrial level and energy metabolism of the heart will be the focus of our follow-up study.

Taken together, these results make it clear that ALM has a positive effect on the improvement of hypertension and demonstrate its clinical promise in the treatment of myocardial hypertrophy caused by hypertension. This study mainly reveals that ALM attenuates oxidative stress and inflammation levels through activation of the PI3K/Akt signaling pathway, thus achieving therapeutic effects in reducing myocardial hypertrophy, revealing the potential of ALM as a drug candidate for the treatment of myocardial hypertrophy characterized by oxidative stress and inflammation.

## Data Availability

All data involved in this study are available from the corresponding author upon reasonable request.
